# Gene Expression Profiles in Parkinson Disease Prefrontal Cortex Implicate *FOXO1* and Genes under Its Transcriptional Regulation

**DOI:** 10.1371/journal.pgen.1002794

**Published:** 2012-06-28

**Authors:** Alexandra Dumitriu, Jeanne C. Latourelle, Tiffany C. Hadzi, Nathan Pankratz, Dan Garza, John P. Miller, Jeffery M. Vance, Tatiana Foroud, Thomas G. Beach, Richard H. Myers

**Affiliations:** 1 Department of Neurology, Boston University School of Medicine, Boston, Massachusetts, United States of America; 2 Bioinformatics Program, Boston University College of Arts and Sciences, Boston, Massachusetts, United States of America; 3 Department of Laboratory Medicine and Pathology, University of Minnesota School of Medicine, Minneapolis, Minnesota, United States of America; 4 Proteostasis Therapeutics, Cambridge, Massachusetts, United States of America; 5 Hussman Institute for Human Genomics, University of Miami Miller School of Medicine, Miami, Florida, United States of America; 6 Department of Medical and Molecular Genetics, Indiana University School of Medicine, Indianapolis, Indiana, United States of America; 7 Banner Sun Health Research Institute, Sun City, Arizona, United States of America; Georgia Institute of Technology, United States of America

## Abstract

Parkinson disease (PD) is a complex neurodegenerative disorder with largely unknown genetic mechanisms. While the degeneration of dopaminergic neurons in PD mainly takes place in the *substantia nigra pars compacta* (SN) region, other brain areas, including the prefrontal cortex, develop Lewy bodies, the neuropathological hallmark of PD. We generated and analyzed expression data from the prefrontal cortex Brodmann Area 9 (BA9) of 27 PD and 26 control samples using the 44K One-Color Agilent 60-mer Whole Human Genome Microarray. All samples were male, without significant Alzheimer disease pathology and with extensive pathological annotation available. 507 of the 39,122 analyzed expression probes were different between PD and control samples at false discovery rate (FDR) of 5%. One of the genes with significantly increased expression in PD was the forkhead box O1 (*FOXO1*) transcription factor. Notably, genes carrying the FoxO1 binding site were significantly enriched in the FDR–significant group of genes (177 genes covered by 189 probes), suggesting a role for FoxO1 upstream of the observed expression changes. Single-nucleotide polymorphisms (SNPs) selected from a recent meta-analysis of PD genome-wide association studies (GWAS) were successfully genotyped in 50 out of the 53 microarray brains, allowing a targeted expression–SNP (eSNP) analysis for 52 SNPs associated with PD affection at genome-wide significance and the 189 probes from FoxO1 regulated genes. A significant association was observed between a SNP in the cyclin G associated kinase (*GAK*) gene and a probe in the spermine oxidase (*SMOX*) gene. Further examination of the *FOXO1* region in a meta-analysis of six available GWAS showed two SNPs significantly associated with age at onset of PD. These results implicate *FOXO1* as a PD–relevant gene and warrant further functional analyses of its transcriptional regulatory mechanisms.

## Author Summary

Parkinson disease (PD) is a neurodegenerative disease, which impairs the motor and cognitive abilities of affected individuals. Although the involvement of specific genes in the disease process has been recognized, the underlying genetic mechanisms are not yet understood. One common investigation approach for PD has been the comparison of gene expression levels in brain tissue from PD cases with those from neurologically healthy controls. We performed such an expression analysis in prefrontal cortex tissue from a set of 27 PD and 26 control samples. One of the 489 differentially expressed genes, forkhead box O1 (*FOXO1*), is involved in transcriptional regulation. Notably, the set of differentially expressed genes identified in our study was enriched for genes regulated by the FoxO1 protein. Analyses of DNA sequence variants known as single-nucleotide polymorphisms (SNPs) in the *FOXO1* region, as well as of PD–relevant SNPs across the genome, suggest functional connections between this gene and 1) the age at onset in PD, and 2) the spermine oxidase (*SMOX*) gene. These findings implicate the involvement of *FOXO1* in PD pathogenesis.

## Introduction

Parkinson disease (PD, OMIM #168600) is a neurodegenerative disorder, which affects primarily motor function (difficulty in movement initiation, tremor, slowness of movement), and secondarily cognitive capabilities of affected individuals. The lifetime risk for the disease is 1.5%, with a median age at onset of 60 and 1.5 increased risk in men compared to women. While a minority of PD cases has been attributed to rare monogenic forms, most cases are likely to be attributed to both genetic and environmental influences [1]. PD has an established pathology, with depletion of up to 60% of dopaminergic neurons in the *substantia nigra pars compacta* (SN) brain region prior to the onset of motor symptoms, and with protein inclusion aggregates known as Lewy bodies. Nevertheless, the specific cellular mechanisms involved in the onset and propagation of PD are still largely undetermined [2].

A common strategy for studying neurodegenerative diseases has been the analysis of gene expression differences between diseased and neurologically healthy control brain samples using microarray technologies. Given its strong pathology in PD, the region of choice for assessing disease-specific expression changes has been SN. Whole SN tissue samples, as well as individually captured dopaminergic neurons from this brain region, have been used in prior microarray studies [3]. Nonetheless, the significant loss of dopaminergic neurons and the likely reactive responses present in surviving neurons at the time of patient death make the interpretation of expression data from SN challenging. The Sutherland et al. study [4] compared results from multiple SN PD microarrays and found low concordance among the implicated genes and pathways. Possible reasons for the inconsistent results might have been the small sample sizes used in individual experiments, the pronounced loss of pigmented SN neurons in PD cases, other types of cellular heterogeneity within and between disease and control specimens, and the large variability attributable to gender, age, RNA quality, post-mortem interval, and co-occurrence of other neurological disorders (e.g. Alzheimer disease pathology). Recently, the Zheng et al. study [3] used a gene-set enrichment meta-analysis approach to analyze expression data from a total of 17 studies (mostly SN, but also studies from other brain regions, as well as blood and human lymphoblastoid cells). They found 10 gene sets to be consistently associated with PD, including the gene set corresponding to 425 *PGC-1α*-responsive nuclear-encoded mitochondrial genes. Given this result and additional expression results from cellular disease models, the authors concluded that *PGC-1α* (*PPARGC1A*, peroxisome proliferator-activated receptor gamma, coactivator 1 alpha, Entrez ID = 10891) is implicated in PD and is a potential therapeutic target for the disease. Additionally, strategies for the integration of different types of data sources for the study of PD have emerged; a recent study by Edwards et al. (2011) combined expression and GWAS data from non-overlapping samples to detect biological pathways that might be relevant for PD [5].

In the current study, we sought to analyze expression differences between PD and neurologically healthy controls in a manner that would maximize our control of possible technical and design confounders, to the extent possible for a tissue homogenate microarray study. Using the One-Color Agilent 60-mer Whole Human Genome Microarray, we investigated expression differences in the prefrontal cortex Brodmann Area 9 (BA9) in the largest PD brain study to date (27 PD and 26 control samples, E-MTAB-812 ArrayExpress dataset). For the microarray experiment we used prefrontal cortex, a brain region which contains dopaminergic neuron projections, does not show the pronounced cell death observed in SN, while still being molecularly and pathologically affected by the disease [2], [6], [7]. The samples included in our study were highly homogenous: all were from males, with high pH values, and none showed significant Alzheimer disease pathology (e.g. the sample is that of pure Lewy body pathology for cases). To our knowledge, this sample is the most homogenous ever studied for PD (Table 1, Table S1). In addition to our microarray expression data, we had genotyping data available for 50 of the 53 samples, consisting of all 56 genome-wide significant SNPs derived from the US-PD GWAS consortium meta-analysis [8]. We combined the 52 genome-wide significant SNPs with minor allele frequencies greater than 0.1 with 189 microarray probes with false discovery rate (FDR) less than 0.05 and located in genes with common FoxO1 regulation in a targeted expression-SNP (eSNP) study. The performed analyses implicate the forkhead box O1 (*FOXO1*) gene as having an important regulatory role for PD. Furthermore, support for *FOXO1* was found in its association to age at onset (AAO) in the US PD-GWAS consortium data [8].

**Table 1:** Description of retained brain samples for the Agilent microarray study.

Sample Type (n)	Age at death, years (range)	PMI^1^, hours (range)	RIN^2^ (range)	Tissue pH^3^ (range)
Control (26)	75.03 (58–97)	13.69 (1.50–39.67)	7.39 (4.8–8.5)	6.66 (6.29–7.32)
PD (27)	77.29 (64–94)	6.47 (1.16–30.75)	7.35 (5.6–8.4)	6.68 (6.43–7.13)

1 PMI: post-mortem interval.

2 RIN: RNA Integrity Number.

3 The pH was measured following a previously established protocol [58].

## Results

**Figure 1:**
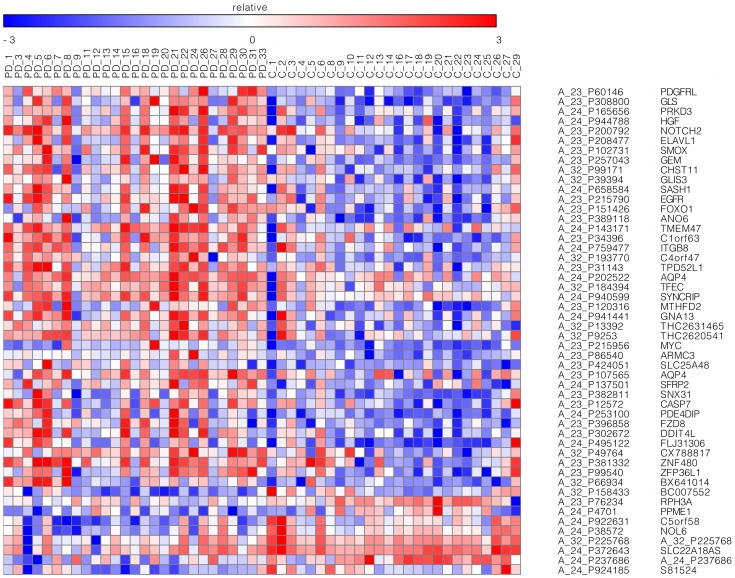
Top microarray probes. Probes with FDR-adjusted p-values smaller than 0.05 and with expression differences between PD and control prefrontal cortex BA9 samples greater than 1.5 fold changes. Twenty-one probes (42%) were in genes with FoxO1 transcription factor binding sites. The GENE-E software (http://www.broadinstitute.org/cancer/software/GENE-E/) was used to generate the heatmap.

Differential expression analysis for 27 PD and 26 control prefrontal cortex samples (see Materials and Methods, Microarray QC and differential expression analysis section) revealed 507 mRNA probes, within 489 expressed regions (known genes, as well as non-genic expressed genomic elements), with FDR-adjusted p<0.05. Among these differentially expressed probes, 50 had fold changes greater than 1.5. These 50 probes are displayed in Figure 1 and all the FDR significant probes are presented in Table S2. Since three of the available microarray samples had RIN values below 6, we performed a secondary differential expression analysis after removal of these samples [9]. The obtained FDR-significant results, consisting of 912 mRNA probes, are displayed in Table S5. The 36 probes that reached FDR-level of significance when the entire set of brains was used, but not after removal of low RIN samples are indicated in Table S2. Notably, the fold changes between PD and control samples were generally small, with few probes having fold changes larger than 2. This result differs from some of the previously published SN studies, where the contrasts between the two groups displayed large fold changes [10], [11], which may be attributable to artifacts introduced in the study of SN.

Functional analyses for the FDR-significant genes that were present in the Database for Annotation, Visualization and Integrated Discovery (DAVID) v6.7 and in Ingenuity's Pathway Analysis software (IPA) were performed. 418 of the 507 FDR-significant probes (82.4%) were mapped to 395 genes present in DAVID's database. None of the gene ontology (GO) terms present in DAVID reached Bonferroni or FDR-adjusted statistical significance at α = 0.05 for this set of genes. Nevertheless, brain-specific GO terms with nominal enrichment were observed, such as “neuron development”, “neuron projection development”, “gliogenesis”, and “neuron differentiation”. The DAVID analysis showed ten transcription factor-binding sites (TFBS) enriched at a Bonferroni level of significance less than 0.001 and with a fold enrichment of at least 1.2 for the mapped FDR-significant genes. The fold enrichment represents the ratio between the percentage of genes in the mapped gene list with a specific TFBS and the percentage of genes in the entire DAVID database with the specific TFBS. Among the ten enriched TFBS, the FoxO1 site (Bonferroni p = 1.3E-4, fold enrichment = 1.4) was the only one that corresponded to a gene that was also differentially expressed at FDR significance in the microarray (Table S2). 177 genes with the FoxO1 TFBS, including *FOXO1* itself, were present among the 395 DAVID mapped genes (44.8%); these genes were covered by 189 FDR-significant probes. Enrichment for the FoxO1 TFBS among FDR significant genes continued to be observed when limiting analysis to only the genes studied in the microarray, and not all genes in the DAVID database (χ^2^ p<2.2E-16, odds ratio = 1.37). Notably, although *PPARGC1A*, gene implicated in the Zheng et al. study [3], was not among the differentially expressed genes in our microarray, this gene was determined to protect dopaminergic neurons when deacetylated by the Sirt1 (sirtuin 1) protein in the MPTP mouse model of PD [12]; *SIRT1* expression was increased in PD samples compared to controls at an FDR-level of significance in our microarray.

The *FOXO1* gene had two different, but strongly correlated (r = 0.75, p = 4.8E-11) probes in the microarray, both with FDR-significantly increased expression in the PD group. Among the FDR-significant probes corresponding to the genes with FoxO1 TFBS (and excluding the two *FOXO1* probes), 78.07% were also associated with an increase in expression for the PD group compared with the control group. This percentage is significantly greater than that observed in the remaining 318 FDR significant probes, where only 66.35% were associated with increased expression in the PD group (χ^2^ = 7.81; p = 0.0052).

We used Ingenuity's IPA software to identify functional categories enriched for significantly associated genes and to build a functional network based on identified categories related to neurological diseases and processes. The network was constructed by starting with 412 unique genes with at least one FDR significant probe, and that were included in the IPA database. The genes present in two of the top nominally enriched functional categories, “Nervous System Development and Function” (individual functions annotation p-values between 2.56E-4 and 2.39E-2) and “Neurological Disease” (individual functions annotation p-values between 5.68E-4 and 2.39E-2), were merged to form a custom network. The *FOXO1* gene was added to this network, and the largest connected component of the network was retained (Figure S1). Among the 31 genes included in this merger of neurologically relevant functional categories (without *FOXO1*), 24 genes with FoxO1 sites were present (77.4%), which is significantly more than the 44.8% observed in all FDR-significant genes.

To validate our microarray results, we used the QuantiGene Plex 2.0 gene expression assay (Affymetrix, Santa Clara, CA, see Materials and Methods, Microarray validation experiment section) for a subset of 8 PD and 9 control samples included in the microarray. We analyzed the expression of 10 genes that contained probes with FDR significance in the microarray. A microarray gene was validated if the fold change obtained for the analysis of the QuantiGene expression data was in the same direction as the fold change obtained for the analysis of the microarray data in the same subset of 17 samples. By this criterion, nine out of the ten considered genes, including *FOXO1*, were validated (Table S3). The difference in expression of *FOXO1* was nominally significant for the validation study.

We compared our results with those obtained using the Affymetrix HG-U133A microarray data published by Zhang et al. [11], which was the only prior PD microarray performed in prefrontal cortex BA9 tissue. With 27 out of the 11,191 genes present on both microarray platforms showing consistent expression dysregulation, we could not detect a significant overlap between the top genes identified by the two BA9 studies (χ^2^ test p = 0.61, see Materials and Methods, Analysis of prior PD prefrontal cortex and substantia nigra microarray studies section). The top genes were defined as the set of genes with FDR adjusted p-values below 0.05 for our Agilent microarray (278 out of the 11,191 genes), and the set of genes with unadjusted p-values smaller than 0.05 for the Affymetrix microarray (1,012 out of the 11,191 genes). Despite the lack of significant overlap between the two studies, *FOXO1* was one of the replicated genes, showing increased expression in PD samples in the Zhang et al. BA9 data (probe = 202723_s_at, p = 0.004, FC = 1.48). The FDR-significant genes from our microarray study with positive nominal signal in the Zhang et al. study are presented in Table S2.

**Figure 2:**
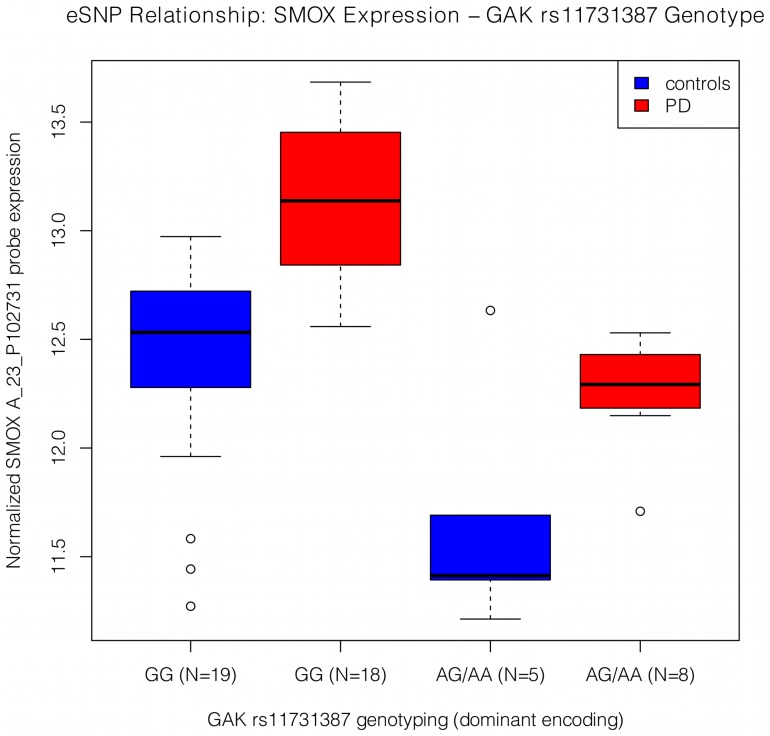
Expression by genotype relationship between the *SMOX* probe, A_23_P102731, and the *GAK* SNP, rs11731387. The box whiskers extend to the most extreme data point, which is at most 1.5 times the interquartile range from the box. The result for the 2-degree of freedom test was p = 8.1E-6, and the eSNP relationship was stronger in PD (p = 7.47E-5, beta = −0.727) than in controls (p = 0.037, beta = −0.494). The minor allele frequency for rs11731387 in the used brain sample was 0.15, and the odds ratio for this SNP in the additive model affection study of the meta-GWAS was 1.35.

To further investigate the observed enrichment for genes containing the FoxO1 TFBS among those identified as FDR significant microarray results, we performed a targeted eSNP analysis in the microarray brain samples. For the eSNP analysis, we evaluated the presence of potential regulatory effects of PD associated SNPs on differentially expressed probes from genes with FoxO1 TFBS (see Materials and Methods, SNP genotyping and eSNP analysis sections). We detected a single eSNP relationship (p = 8.1E-6) that met the Bonferroni corrected p-value threshold of 5.36E-5 for the used effective number of SNPs (Figure 2). This finding involved a PD GWAS genome-wide significant SNP, rs11731387, present intronically in the *GAK* gene (cyclin G associated kinase, Entrez ID = 2580) and a probe present in the 3′-UTR of the *SMOX* gene (spermine oxidase, Entrez ID = 54498). The rs11731387 minor allele was associated with higher risk for PD in the US PD-GWAS consortium meta-analysis (p = 8.81E-9, beta = 0.3018, [8]) and with decreased *SMOX* expression. Although stronger in the PD subgroup, the eSNP relationship was present in both PD (p = 7.47E-5, odds ratio = −0.727) and controls (p = 0.037, odds ratio = −0.494). *SMOX* probe expression was increased in the PD group. Interestingly, *SMOX* is a gene involved in the dopamine receptor signaling pathway, which is a process that has had evidence for involvement in PD [13], [14], [15].

Additionally, we tried to reduce the group of FDR significant, FoxO1 TFBS genes to a subset of genes which act as mediators for the relationship between *FOXO1* expression and disease status (see Materials and Methods, Mediation analysis section). Twenty-nine genes, including *SMOX*, showed evidence that suggested they may act as mediators for the *FOXO1* effect on PD (Table 2). Therefore, these genes may be most relevant to the *FOXO1* pathway relating to PD, and could be important gene candidates for further analyses of *FOXO1* involvement in the disease.

**Table 2:** FoxO1 TFBS genes with evidence of partial mediation for the relationship between *FOXO1* and PD.

Gene	Probe	Direct effect^1^	Indirect effect^1^	Ratio of indirect and direct effects	% total effect mediated	p-value
*ARMC3*	A_23_P86540	0.63	0.68	1.09	52.1%	0.037
*SMOX*	A_23_P102731	0.62	0.54	0.86	46.3%	0.032
*NOTCH2*	A_23_P200792	0.62	0.46	0.73	42.2%	0.030
*CDKAL1*	A_23_P73058	0.83	0.53	0.64	39.0%	0.035
*PTPN13*	A_23_P18493	0.77	0.46	0.59	37.2%	0.043
*SLC27A1*	A_23_P131111	0.84	0.48	0.57	36.3%	0.030
*ZNF438*	A_23_P161156	0.70	0.39	0.56	36.0%	0.026
*PARD3*	A_24_P35478	0.80	0.45	0.56	36.0%	0.040
*KDSR*	A_32_P515088	0.72	0.40	0.55	35.5%	0.045
*ANAPC16*	A_32_P32207	0.77	0.42	0.54	35.2%	0.036
*LGR4*	A_24_P90216	0.74	0.38	0.52	34.2%	0.029
*EIF4G3*	A_23_P126241	0.72	0.36	0.50	33.5%	0.032
*TOR1AIP2*	A_24_P839239	0.78	0.38	0.49	33.0%	0.041
*ERLIN1*	A_23_P202029	0.85	0.42	0.49	33.0%	0.040
*MAPRE1*	A_24_P220058	0.79	0.37	0.46	31.6%	0.042
*BCL2*	A_23_P352266	0.78	0.36	0.46	31.4%	0.041
*GLIS3*	A_32_P39394	0.77	0.35	0.45	31.1%	0.037
*EZR*	A_23_P19590	0.82	0.36	0.44	30.5%	0.048
*INPPL1*	A_23_P36322	0.84	0.36	0.42	29.8%	0.039
*ZC3H12C*	A_23_P388993	0.83	0.35	0.42	29.6%	0.038
*ELAVL1*	A_23_P208477	0.79	0.33	0.41	29.2%	0.045
*ATP6V0E1*	A_23_P213840	0.86	0.34	0.39	28.1%	0.042
*SOX2*	A_23_P401055	0.83	0.32	0.39	27.9%	0.047
*FZD7*	A_23_P209449	0.94	0.35	0.37	27.0%	0.046
*AGFG2*	A_23_P311640	0.81	0.29	0.36	26.7%	0.046
*AQP4*	A_23_P107565	0.92	0.33	0.35	26.2%	0.043
*SLITRK1*	A_23_P37041	0.84	0.29	0.34	25.4%	0.047
*NECAB3*	A_23_P68628	0.90	0.29	0.32	24.4%	0.050
*ACSS3*	A_23_P339119	0.85	0.27	0.31	23.9%	0.048

1 The direct effect represents the effect of *FOXO1* expression on PD directly, while the indirect effect represents the effect that is mediated through each FoxO1 TFBS gene.

Finally, we analyzed genome-wide SNP data from the US PD-GWAS consortium meta-analysis [8] to further investigate the role of SNPs in the *FOXO1* region for PD affection or age at onset (AAO). While no SNP in the *FOXO1* region reached the required p-value for significance of 8.68E-5 in the affection meta-analysis (see Materials and Methods, PD affection and age at onset meta-analysis for the FOXO1 gene region section), two SNPs in the region reached this level of significance in the AAO meta-analysis (Table 3). AAO data were available for most PD brains in the microarray, so we investigated the *FOXO1* probe expression – age at onset relationship (Table S1), while adjusting for age at death, post-mortem interval (PMI), and RNA integrity (RIN). *FOXO1* expression was not significantly associated with age at onset (A_23_P151426: p = 0.46, beta = 0.008; A_24_P22079: p = 0.90, beta = −0.0007).

**Table 3:** Top age at onset (AAO) meta-analysis results for the *FOXO1* region.

SNP	Position^1^	A1/A2	A1 freq	Beta AAO	p-value AAO^2^	Direction of effect in AAO study^3^	Imp/geno^4^	Beta affection^5^	p-value affection^5^	SNPExpress eSNP results beta/p/probe type^6^
rs4509910	40887620	T/G	0.3931	1.1196	**3.14E-5**	+++?++	IIIIIG	0.0347	0.329	N/A
rs9532809	40889256	T/C	0.2168	1.4636	**4.57E-5**	?++?++	IIIIIG	0.0243	0.606	N/A
rs7987856	40890286	T/C	0.3214	1.0053	5.32E-4	−++?++	IIGIGI	0.0219	0.566	98.91/3.5E-3/exon (40,028,022–40,031,008^1^)
rs1782791	39876659	A/G	0.2451	−0.9411	5.96E-4	+−−−−−	IIIIIG	−0.0207	0.585	N/A
rs7335637	39876115	A/G	0.2449	−0.9357	6.56E-4	+−−−−−	IIIIII	−0.0209	0.582	N/A
rs17061200	39841522	T/C	0.3818	−0.8323	7.69E-4	−+−−−−	IIIIIG	−0.0076	0.823	N/A
rs927924	39883468	A/C	0.5911	0.7887	9.26E-4	++++++	GGGGGG	0.0548	0.095	p>0.05
rs11617666	39879204	T/C	0.2357	0.9169	9.73E-4	−+++++	GGGIGI	0.0256	0.505	p>0.05

1 Position for genome build 36.3 (hg18).

2 Required p-value level for significance p-values = 8.68E-5; **bold** indicates significant p-value.

3 Direction of effects are listed in the following order: PROGENI/GenePD, NIA Phase I, NIA Phase II, LEAPS, HIHG, NGRC; a question mark (?) indicates that the marker failed imputation (Rsq<0.30).

4 Values for imputed (I) or genotyped (G) status.

5 The meta-analysis included the PROGENI/GenePD, NIA Phase I, NIA Phase II, HIHG, and NGRC studies.

6 The SNPExpress database [29] was used to look up eSNP relationships between the expression of *FOXO1* transcripts or exons and the SNPs of interest. The best *FOXO1* eSNP result is displayed if a p-value<0.05 was observed. The presented eSNP results were obtained in brain tissue. N/A values are used when the SNPs were not available in the database.

## Discussion

We performed a microarray study in prefrontal cortex Brodmann Area 9 (BA9), in a set of homogenous (male, non-significant Alzheimer disease pathology) and high quality (high pH, good RNA integrity) PD and control brain samples. To our knowledge, this is the largest and most uniform microarray brain study to date in PD, and we expect the expression data and available covariate information to represent an invaluable resource for the PD community (ArrayExpress E-MTAB-812 dataset). While the microarray was not performed in the most-involved brain region in PD, the *substantia nigra pars compacta* (SN), we propose that the use of prefrontal cortex tissue, or of other brain regions with neuropathological involvement of disease, but reduced neuronal cell death, has the potential to overcome limitations associated with the use of severely disease affected tissues; this is especially the case when whole tissue homogenate samples are considered. While the SN is almost completely depleted of dopaminergic neurons by the time of autopsy [16], prefrontal cortex tissue does not show such dramatic neuronal death. Nevertheless, prefrontal cortex is very frequently neuropathologically involved in PD [7] (74% of the cases investigated in the Beach et al. study showed Lewy bodies and associated fibers in this brain region), and shows biochemical alterations related to the disease process [2], [17]. Since the Lewy bodies and associated fibers appear later during the disease in the prefrontal cortex [16], the study of BA9 may reveal pathogenically relevant disease changes.

This study found evidence of a significant role for the transcription factor gene *FOXO1* and genes under its transcriptional regulation: (1) *FOXO1* expression was significantly increased in PD samples in our study, and (2) the top microarray results were enriched for genes containing the FoxO1 transcription factor-binding site. The increased *FOXO1* expression in PD samples is consistent with a previous PD BA9 microarray study reported by Zhang et al. [11], the only prior PD expression study performed in this brain region. The majority of prior PD microarray studies performed in SN tissue also reported increased *FOXO1* expression and enrichment of FoxO1 TFBS genes in their top results, with significant meta-analysis p-values for the two *FOXO1* probes present on the Affymetrix HG-U133A chip ranging from 4.1E-3 to 3.2E-4 (Table S4).

To further explore the significance of FoxO1 TFBS enrichment, we performed a targeted eSNP study for FDR-significant microarray probes located in genes with FoxO1 regulation and SNPs known to be associated with PD affection at genome-wide level of significance [8]. This analysis revealed a significant relationship between the *GAK* SNP rs11731387 and probe A_23_P102731 in the *SMOX* gene. The rs11731387 minor allele was associated with both increased PD risk and decreased *SMOX* expression. Given the observed increase in *SMOX* expression in PD compared to control samples, we propose that elevated *SMOX* expression in the brain is required as a protective mechanism against the biochemical changes that lead to and are present in PD, and is not a direct cause of the disease. This would explain why a SNP that prevents sufficiently elevated *SMOX* expression levels could enhance sensitivity to PD. When both the *GAK* SNP and the expression of the A_23_P151426 *FOXO1* probe were included as predictors for the expression of the *SMOX* probe, the magnitude of the effect for these two predictors changed only slightly from the initial results, both of them remaining significant. Given this evidence, we propose that *FOXO1*, *GAK*, and *SMOX* are involved in a common biological pathway, with *FOXO1* and *GAK* independently influencing *SMOX* expression and, consequently, PD risk.

Multiple sources of evidence have recently implicated the cyclin G associated kinase (*GAK*) gene in PD [8], [18], [19], [20], [21], [22], [23], although it has been unclear how this gene influences the disease. The SMOX enzyme plays a role in polyamine catabolism, and it is known to be involved in the response to drugs, stressful stimuli, and apoptosis. High expression levels of *SMOX* have been found in the brain and the polyamine catabolism system has been implicated in psychiatric conditions [24]. Notably, the SMOX protein is a component of the dopamine receptor signaling pathway, where, together with the MAOA (monoamine oxidase A), MAOB (monoamine oxidase B), and IL4I1 (interleukin 4 induced 1) proteins, it makes up the MAO complex (Ingenuity Knowledge Base). Although inconsistently, *MAOA* and *MAOB* have been linked to PD in several genetic studies [15], [25], [26], [27], [28], and the dopamine receptor signaling pathway has been implicated in the disease [13], [14], [15].

Further, we performed a mediation analysis to determine if the expression of any of the FDR-significant FoxO1 TFBS genes shows evidence to potentially act as an intermediary step for the observed relationship between *FOXO1* expression and PD case/control status. This analysis showed the expression values of 29 genes (Table 2) to act as mediating variables. Notably, *SMOX* was one of these genes, with the second highest percentage of total mediated effect. This set of 29 genes is of particular interest for future studies of *FOXO1* involvement in PD.

Finally, we investigated the *FOXO1* region for SNPs linked to PD affection or PD age at onset (AAO) using imputed data from the US PD-GWAS consortium [8]. While no SNP reached statistical significance for affection, two SNPs from the *FOXO1* region, rs4509910 and rs9532809, were significantly associated with increased PD AAO. The association results for these two SNPs and six additional ones with p-values<1E-3 for the AAO analysis are displayed in Table 3. Using the SNPExpress database [29], we tried to determine whether or not there is evidence for any relationship between these SNPs and *FOXO1* expression in brain tissue. Only three of the SNPs in Table 3 were present in the SNPExpress database; the two SNPs with significant AAO association were not among them. From the three SNPs present in the database, rs7987856 showed evidence for association with *FOXO1* expression: its minor allele was associated with increased expression of the 3′-UTR exon of the *FOXO1* NM_002015 transcript (beta = 98.91, p = 3.5E-3). This SNP is in high LD with the top AAO SNP, rs4509910, with an R^2^ of 0.76 in the release 22 of the HapMap CEU population, as determined in Haploview [30]. This result might be indicative of the existence of alternative *FOXO1* transcripts in the brain, which could have an effect on the progression of PD.

The Forkhead box, subgroup O (FOXO) transcription factors have been implicated recently in studies of known PD genes and aspects of PD neurodegeneration. The FOXO3a protein was determined to control *PINK1* (PTEN induced putative kinase 1, Entrez ID = 65018) transcription in mouse and human cells subjected to growth factor deprivation [31], and it was found to localize to Lewy bodies and Lewy neurites [32]. Additionally, the homologues of human *FOXO1* have been recently involved in *Drosophila melanogaster* or *Caenorhabditis elegans* models of PD, with different post-translational modifications of the protein showing either protective or harmful effects. *Drosophila PINK1* null mutants display mitochondrial dysfunction and dopaminergic neuron loss. Koh et al. [33] showed that Sir2 (the homologue or the human SIRT1/sirtuin 1 protein) and FOXO protect mitochondria and dopaminergic neurons downstream of PINK1. Sir2 and SIRT1 are deacetylases, which have FOXO and FoxO1, respectively, as one of their targets. This protective effect was observed to take place through overexpression of two FOXO target genes, *SOD2* (superoxide dismutase 2, mitochondrial) and *Thor*. Kuwahara et al. recently [34] studied the role of Serine-129 phosporylation of α-synuclein in the transgenic *C. elegans* (Tg worm) model of synucleinopathy. The pan-neuronal overexpression of nonphosphorylatable (S129A) α-synuclein showed severe defects in the Tg worm. Gene expression profiling of S129A-Tg worms showed strong upregulation of Daf-16/FOXO pathway genes, which the authors proposed to act against the dysfunction caused by the S129A-α-synuclein. Two additional studies [35], [36] reported that phosporylation of FOXO at the same amino acid residue by the LRRK2 (leucine-rich repeat kinase 2) protein or the PRKG2 (protein kinase, cGMP-dependent type II) protein in *Drosophila* reduces dopaminergic neuron survival.

With our current study, we bring further evidence for the importance of the *FOXO1* gene in PD. In addition to the differential expression of this gene in PD versus control BA9 and SN tissue, an increased number of genes under the transcriptional regulation of the FoxO1 transcription factor also have altered expression in BA9 tissue in our study. Finally, SNPs in the *FOXO1* region are associated with the age at onset for PD. The results of our study warrant further investigation of the *FOXO1* gene and of its protein product in the pathogenesis of PD, and we consider the exploration of the relationship between *FOXO1*, *SMOX*, and *GAK* in various PD models as a possible follow-up step. Although we presented multiple sources of evidence for the involvement of *FOXO1* in PD, we cannot rule out that the change in *FOXO1* expression may be a secondary effect seen mainly in prefrontal cortex and that this may not be primarily involved in the pathogenesis of PD.

While *FOXO1* represents our main finding, additional genes with FDR-significant microarray probes and prior evidence for involvement in PD analyses are worth mentioning. A few of these genes are: *HGF* (hepatocyte growth factor, Entrez ID = 3082), which encodes a protein that promotes the survival and migration of immature neurons [37], [38], *SLC41A1* (solute carrier family 41, member 1, Entrez ID = 254428), which was recently implicated in PD genome-wide association and genotyping studies [39], [40], *EGFR* (epidermal growth factor receptor, Entrez ID = 1956), a gene shown to play a crucial role in the dopamine-induced proliferation of adult neural precursor cells of subgranular, subventricular, and subependymal zones [41], [42], *AQP4* (aquaporin 4, Entrez ID = 361), which encodes for the predominant aquaporin found in the brain, water channel involved in the pathophysiology of cerebral disorders [43], and *NEDD4* (neural precursor cell expressed, developmentally down-regulated 4, Entrez ID = 4734), a gene that encodes for a ubiquitin ligase involved in the endosomal-lysosomal pathway and ubiquitinates alpha-synuclein [44]. These and other genes with prior evidence for involvement in PD-related processes are promising targets for further studies.

Finally, it is worthwhile to note the lack of overlap that we observed between our study and the BA9 study performed by Zhang et al. [11]. Some of the observed inconsistency may be due to significant differences between these two microarray analyses. For example, the different microarray platforms might assess different transcripts for the considered genes, gender and disease pathology might have a significant impact on the expression levels of a large number of genes, and the different available sets of covariates might affect the expression results (e.g. RIN is not available for the Zhang et al. data). Even with this apparent lack of overlap, we believe that transcriptome data are relevant and can help bring significant insights in the study of PD. A possible way to alleviate incoherent results could be the establishment of standard protocols for expression studies in brain samples, which is an important, yet overlooked objective. Nonetheless, those findings that do replicate, even with the existent microarray data (e.g. *FOXO1*), may be pointing to important disease-related pathways.

## Materials and Methods

### Microarray samples

Brain tissue from the prefrontal cortex Brodmann Area 9 (BA9) was obtained from three different brain banks: the Harvard Brain Tissue Resource Center McLean Hospital, Belmont, Massachusetts, the Human Brain and Spinal Fluid Resource Center VA, West Los Angeles Healthcare Center, California, and the National Brain and Tissue Resource for Parkinson's Disease and Related Disorders at Banner Sun Health Research Institute, Sun City, Arizona [45]. Thirty-three Parkinson disease (PD) and 29 control samples were selected for the microarray study. The samples were selected based on the following criteria: (1) no significant Alzheimer disease pathology (specified by neuropathology reports), (2) tissue pH>6.25, (3) similar ages of death for PD cases and controls, and (4) male.

### Microarray QC and differential expression analysis

Total RNA for the 33 PD and 29 control samples was extracted with TRIzol (Invitrogen, Carlsbad, CA). RNA was purified using the RNeasy MinElute Cleanup columns (Qiagen Sciences Inc, Germantown, MD) and its quality was assessed with an Agilent Bioanalyzer Nano Chip 2100 (Agilent, Foster City, CA). 1.65 µg of each RNA sample were labeled and hybridized to the One-Color Agilent 60-mer Whole Human Genome Microarray (#G4112A) at the Agilent Microarray Facility of the Whitehead Institute for Biomedical Research (Cambridge, MA). The dye-normalized and post surrogate processed signal for the green channel, gProcessedSignal, obtained from Agilent's Feature Extraction Software was used for downstream analyses. The raw expression data for the 62 samples were evaluated for individual array quality (MA plots), array intensity distributions (box plots and density plots) and between-array differences (heat maps representing the distance between arrays) using the arrayQualityMetrics Bioconductor package. Nine outlier samples were detected based on the arrayQualityMetrics default criteria [46] and were dropped from further analyses. Table 1 describes the retained microarray samples. Post-mortem interval was the only significantly different covariate between the retained cases and controls (p = 0.02).

Microarray probes were removed if they had expression values outside the detectable spike-in range in more than 50% of the control arrays and more than 50% of the PD arrays, or if they had any of the Agilent flags IsWellAboveBG = 0, gIsSaturated = 1, gIsFeatPopnOL = 1, gIsFeatNonUnifOL = 1 in more than 75% of the arrays. The median expression value was used for replicated probes that passed the above filtering criteria. A total of 39,122 probes out of the total 45,015 probes present on the microarray chips were analyzed in the expression and eSNP studies. The expression data for the retained probes of the 53 arrays (E-MTAB-812 ArrayExpress dataset) were quantile normalized, and the obtained values were base 2 logarithm transformed. All the microarray processing analyses were performed in R (http://www.R-project.org), using the Agi4x44PreProcess and the limma Bioconductor packages.

The relationship of PD/control status to probe expression levels was determined using linear regression in R. The normalized and log 2 transformed mRNA levels were modeled as the dependent variable and the association of PD/control status was adjusted for RNA integrity (RIN), post-mortem interval (PMI) and age at death. The RIN and pH were the most highly correlated variables in our data (Spearman correlation coefficient = 0.403, p-value = 0.001), and we decided to include in the linear regression model only one of these two variables, to avoid the problem of over-adjustment. We chose the RIN variable, given its larger range of values compared with pH (Table 1). False discovery rate (FDR) adjustment was applied to the obtained p-values for the PD/control-probe expression relationship to account for multiple comparisons.

### DAVID analysis

The Agilent identifiers of the FDR significant probes were uploaded and mapped to genes in the Database for Annotation, Visualization and Integrated Discovery (DAVID v6.7, http://david.abcc.ncifcrf.gov/, [47], [48]) for functional annotation. All available functional categories were considered, including Gene_Ontology, Pathways, and Protein_Interactions (contains the transcription factor binding site data from the UCSC database).

### Ingenuity Pathway Analysis

The genes corresponding to FDR significant microarray probes were analyzed through the use of Ingenuity Pathways Analysis (Ingenuity Systems, www.ingenuity.com). A data set containing FDR significant Agilent probe identifiers and corresponding fold changes was uploaded into the application. Each identifier was mapped to its corresponding gene in the Ingenuity Knowledge Base. These genes were overlaid onto a molecular network developed from information contained in the Ingenuity Knowledge Base. A network of genes with involvement in neurological diseases and processes was created (Figure S1).

### Microarray validation experiment

The QuantiGene Plex 2.0 gene expression assay was used for the validation of the microarray (Affymetrix, Santa Clara, CA). The expression levels of 10 genes containing microarray probes with FDR-adjusted p-values smaller than 0.05 (Table S3) and of two control genes (*TUBG1*, tubulin, gamma 1; *HPRT1*, hypoxanthine phosphoribosyltransferase 1) were evaluated in a subset of 8 PD and 9 control samples from the Agilent microarray experiment (Table S1). The QuantiGene probes designed by Affymetrix targeted the exact transcripts as the ones targeted by the considered Agilent probes (as defined by transcripts present in the UCSC Genes, RefSeq Genes, and Ensembl Gene Predictions tracks from the UCSC genome browser). Gene expression measurements were performed in triplicates in lysed brain tissue, without prior RNA extraction (the RIN covariate was not available). To evaluate the gene expression differences between the PD and control samples, the following procedure was used: 1) for each gene expression measurement, the background value was extracted from the raw expression count; 2) given the 3 different background-extracted expression measurements for each gene, in each sample, average expression values were calculated; 3) the mean expression values for the ten genes were normalized by the geometric mean of the two control genes in each sample; 4) the base 2 logarithm of the obtained normalized values was calculated; 5) a linear model that included age and PMI was used to determine the difference in expression between the two groups.

### Analysis of prior PD prefrontal cortex and substantia nigra microarray studies

The prefrontal cortex Brodmann Area 9 (BA9) microarray expression data published by Zhang et al. [11] were used as a replication study for our microarray results. The Affymetrix CEL files for 14 PD and 16 control samples (Affymetrix Human Genome U133A Array) and the corresponding annotation file were downloaded from ArrayExpress (http://www.ebi.ac.uk/arrayexpress/, E-GEOD-20168). The gcrma method was used to background correct, normalize, and summarize probes for the 30 brain samples. The obtained normalized and base 2 logarithm transformed expression values for the 22,283 available probes were modeled as the dependent variable and the association of PD/control status was adjusted for sex, age at death, PMI, and pH. One control sample lacked covariate information and was removed (GSM506036_1134_BA9_Cm.CEL). After adjustment for covariates, none of the analyzed probes reached FDR significance. To compare our Agilent microarray results with the Zhang et al. Affymetrix results, we annotated the probes for the two microarrays, and assigned the probe with the best p-value to each gene. For the Affymetrix data, 17,564 of the available probes could be assigned to 11,441 Entrez identifiers, while for the Agilent data, 29,927 probes could be assigned to 20,474 Entrez identifiers. There were 11,191 Entrez identifiers common for the two microarrays, and we used a χ^2^ test to evaluate if the overlap between the top genes observed in the two microarrays was larger than expected by chance. For the purpose of the χ^2^ test, we defined the top genes as the set of FDR significant genes (FDR adjusted p-value<0.05) for our Agilent microarray [278 genes with Entrez IDs and in common with the Affymetrix array], and the set of genes with unadjusted p-values smaller than 0.05 for the smaller Affymetrix microarray [1,012 genes with Entrez IDs and in common with the Agilent array]. The overlap between these two sets of genes consisted of 27 genes, which are highlighted in Table S2.

Additional Affymetrix PD expression studies performed in the *substantia nigra* (SN) brain region and present in the ArrayExpress or in the National Brain Databank (NBR, http://national_databank.mclean.harvard.edu/brainbank/Main) public repositories were analyzed similarly to the Zhang et al. data. Since the available covariates for each of the studies varied, we present the results obtained when 1) no covariate was added to the used linear model, and 2) the covariates age or sex (depending on availability) were included. Only the expression studies containing at least one of the two *FOXO1* probes present in the BA9 Affymetrix study, 202723_s_at and 202724_s_at, were considered (Table S4). This includes PD studies performed on the following Affymetrix chips: HG-U133A, HG-U133_Plus_2, and HG-Focus (E-GEOD-8397, E-GEOD-20163, E-GEOD-20164, E-GEOD-20186, E-GEOD-20295, E-GEOD-7621, E-GEOD-20141, E-GEOD-20333, and the Simunovic et al. PD study [10] present in NBR). We meta-analyzed the results obtained using 1) no covariates and 2) the covariates age or sex (depending on availability) for the 3 *FOXO1* probes included in all or part of the 9 studies using the weighted Z-score approach [49]. This method was chosen since it takes into account both the direction of association and the sample size of the individual studies. The Z-scores for the microarray probes of each expression study were obtained from p-values using the standard normal distribution. This conversion was performed in R by using the function qnorm(p-value/2) and changing the sign of the Z-statistic to match the direction of the estimate of association.

### Mediation analysis

A mediation analysis was performed to assess whether or not the observed association between PD and *FOXO1* expression acted through a pathway containing any of the FDR significant FoxO1 TFBS genes. Mediation is assessed by a multistep analysis [50], in which the total effect of *FOXO1* is broken down into a direct effect and an indirect effect, acting through the intervening gene. The three analysis steps were: 1) in order to decompose the effects, a logistic regression was performed with PD as the dependent variable and the more strongly associated *FOXO1* probe (A_23_P151426) as the predictor to establish the total effect (in the original microarray analysis, expression was used as the dependent variable); 2) a linear regression was performed to establish association between *FOXO1* expression and the expression of each of the PD-associated FoxO1 TFBS genes; 3) a logistic regression was performed using each of the FoxO1 TFBS genes as a predictor of PD including *FOXO1* expression in the model. All regressions were adjusted for age, PMI, and RIN. The direct effect is determined from the beta estimate of *FOXO1* in step 3 of the analysis, while the indirect effect is the product of the beta estimates for the relation between *FOXO1* and the FoxO1 TFBS gene and the relation between the TFBS gene and PD after standardization of the betas to account for combination of linear and logistic regressions [51]. Finally, the null hypothesis that the indirect effect equals zero is tested using a Z test [52]. The results are displayed in Table 2.

### SNP genotyping

The 53 retained microarray samples were included among 5,849 PD cases and controls genotyped in the US PD-GWAS consortium meta-analysis replication sample [8]. The samples were genotyped using a custom Illumina genotyping array of 768 SNPs, and 56 SNPs provided genome-wide level of significance in the combined discovery and replication phases, and were considered for functional eSNP analyses of the microarray data. Three of the microarray brain samples failed to genotype at the accepted 98% success rate and were removed from the eSNP analysis.

### eSNP analysis

We performed a targeted *trans*-effect eSNP analysis in the microarray brain samples for: 1) 52 of the 56 genome-wide significant SNPs from the US PD-GWAS consortium study with minor allele frequencies (MAF) of at least 0.1, and 2) a set of 189 microarray probes with FDR-adjusted p-values<0.05 and which mapped to genes with FoxO1 TFBS (Table S2). Many of the genome-wide significant SNPs in the PD associated regions were in strong to moderate linkage disequilibrium (LD); therefore, we used the program SimpleM [53] to determine the effective number of SNPs tested after accounting for LD in each of the different regions to be N = 34. A modified Bonferroni correction method [54] was used to calculate the required eSNP p-value for a 0.05 alpha level as 5.36E-5. Association between the SNPs and probe expression levels was evaluated in the 26 cases and 24 controls using a 2-degree of freedom (df) linear regression model implemented in Plink [55]. The 2-df model permits a simultaneous test of association between genotype and expression and between genotype and difference in association between cases and controls. This method has been used previously for eQTL studies involving mixed case and control samples and increases the power to detect effects of SNPs on expression levels that may be unique to disease [56]. In addition to including SNP, case status and the SNP x case status interaction term, the linear models were adjusted for RNA integrity number (RIN), post-mortem interval (PMI), and age at death. All SNPs were coded using a dominant model.

### PD affection and age at onset meta-analysis for the FOXO1 gene region

We considered the region on chromosome 13 covering the *FOXO1* gene, as well as the areas up to 1 Mb away from the 3′ and 5′ ends of the gene (chr13: 39,027,801–41,138,734, hg18). In this region, there were 2,103 imputed SNPs present in the Pankratz et al. [8] meta-analysis of PD affection. Using the SimpleM program [53] and the imputed data for the NGRC GWAS, the largest study included in the meta-analysis, we determined the corresponding number of effective SNPs in the *FOXO1* region to be N = 576. Since SimpleM uses only genotype data, each SNP was assigned the imputed genotype with the highest confidence for the purpose of this analysis. Given this number of effective SNPs, a p-value of 8.68E-5 was required for an alpha level of 0.05. In addition to the PD risk meta-analysis conducted by the US PD-GWAS consortium [8], a meta-analysis of age at onset of PD was conducted in 6 PD GWAS studies: the five studies present in the US PD-GWAS consortium (PROGENI/GenePD, NIA Phase I, NIA Phase II, HIHG, NGRC) and the LEAPS study [57]. Prior to meta-analysis, results were filtered for imputation efficiency and any study with a MACH-derived Rsq<0.30 did not contribute a result for that SNP to the meta-analysis. Meta-analysis was performed with METAL ([49], http://www.sph.umich.edu/csg/abecasis/Metal/) using an inverse-variance weighting scheme. This allowed an overall effect size to be estimated. Genomic control was employed so that results were down-weighted if the study's lambda exceeded 1.00.

## Supporting Information

Figure S1 IPA generated network enriched in neurologically involved genes. All displayed genes contain at least an FDR-significant probe in the Agilent microarray study. Up-regulation in PD compared to control samples is represented with red color, and down-regulation with green color. The genes depicted as ovals contain FoxO1 transcription factor binding sites, while the genes depicted as triangles do not. Solid lines correspond to direct interactions, and dashed lines to indirect interactions.
(TIF)

Table S1 Full description of microarray sample.
(DOC)

Table S2 Agilent microarray FDR-significant probes. ^1^The probe and gene names corresponding to genes with FoxO1 TFBS are displayed in bold. The probes that do not reach FDR-level of significance when the 3 samples with RIN<6 are dropped from the differential expression analysis are highlighted in red. ^2^The p-values of the genes that had at least one probe with nominal significance (p<0.05) in the Zhang et al. study are displayed in bold.
(XLSX)

Table S3 Validation study results.
(DOC)

Table S4 *FOXO1* probe expression results for all Affymetrix *substantia nigra* PD microarrays available in the Array Express and National Brain Databank repositories^1^.
(DOC)

Table S5 Agilent microarray FDR-significant probes after removal of low RIN samples. ^1^The probe and gene names corresponding to genes with FoxO1 TFBS are displayed in bold.
(XLSX)
