# Supplementary figures and images for: Gene Expression Profiles in Parkinson Disease Prefrontal Cortex Implicate FOXO1 and Genes under Its Transcriptional Regulation
Source: PLoS Genet. 2012 Jun 28;8(6):e1002794. doi: 10.1371/journal.pgen.1002794 (PMC3386245; doi:10.1371/journal.pgen.1002794)

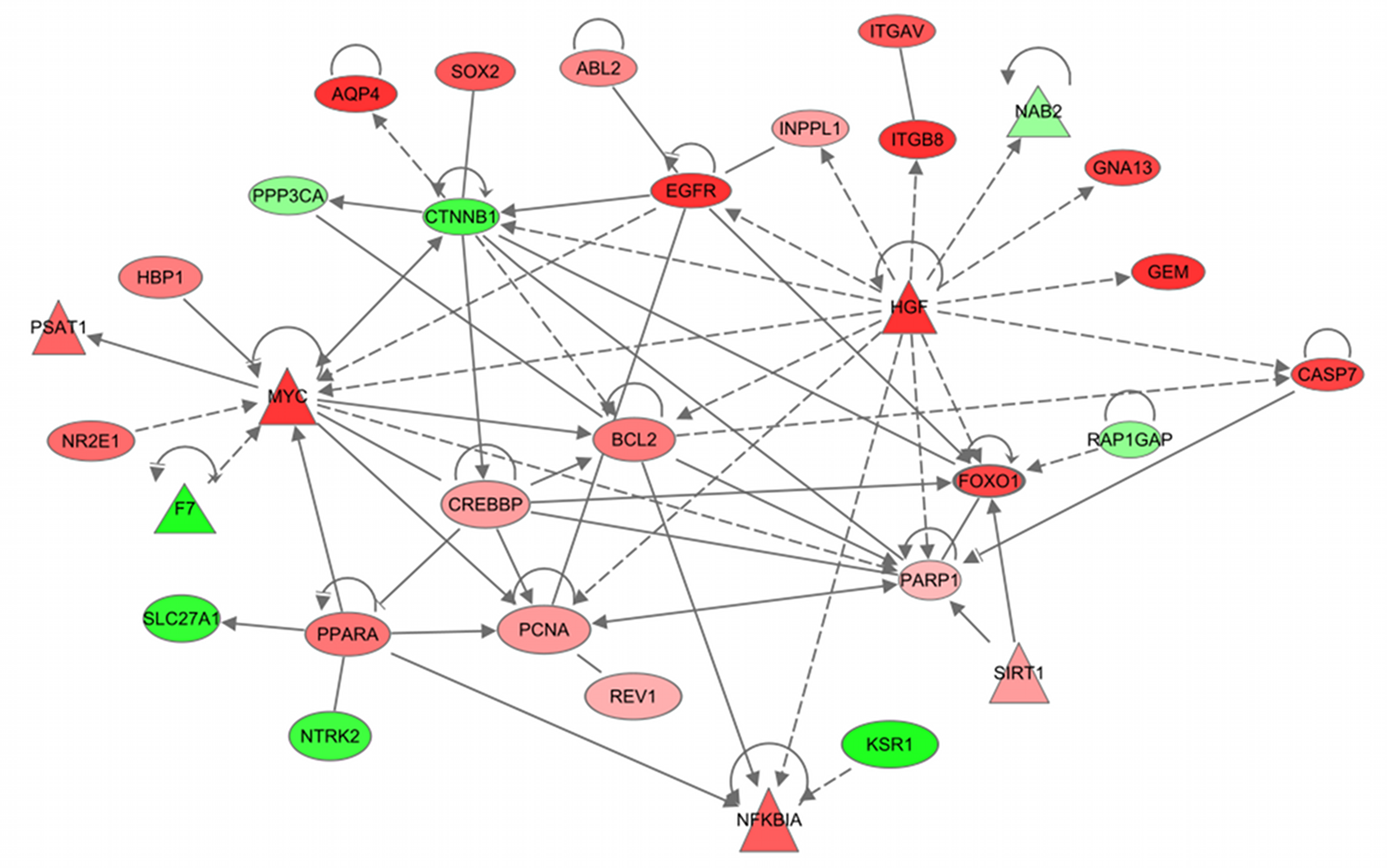

Supplement: Figure S1 — IPA generated network enriched in neurologically involved genes. All displayed genes contain at least an FDR-significant probe in the Agilent microarray study. Up-regulation in PD compared to control samples is represented with red color, and down-regulation with green color. The genes depicted as ovals contain FoxO1 transcription factor binding sites, while the genes depicted as triangles do not. Solid lines correspond to direct interactions, and dashed lines to indirect interactions. (TIF) [file pgen.1002794.s001.tif]
