# Supplementary material for: Gene Expression Profiles in Parkinson Disease Prefrontal Cortex Implicate FOXO1 and Genes under Its Transcriptional Regulation
Source: PLoS Genet. 2012 Jun 28;8(6):e1002794. doi: 10.1371/journal.pgen.1002794 (PMC3386245; doi:10.1371/journal.pgen.1002794)
Supplement: Table S1 — Full description of microarray sample. (DOC) [file pgen.1002794.s002.doc]

Supplementary Table 1. Full description of microarray sample.

| Type | Brain sample^1^ | Age at death | PMI | pH | RIN | Age at onset | Braak stage^2^ | Cortex Lewy bodies^2^ |
| --- | --- | --- | --- | --- | --- | --- | --- | --- |
| Control | **C_1** | 86 | 3 | 6.43 | 6.7 | N/A | I | no |
|  | **C_2** | 73 | 2.25 | 6.62 | 5.5 | N/A | III | no |
|  | C_3 | 91 | 1.5 | 6.29 | 7.1 | N/A | II | no |
|  | **C_4** | 82 | 2.16 | 6.64 | 8 | N/A | III | no |
|  | **C_5** | 97 | 1.5 | 7.13 | 8.1 | N/A | III | no |
|  | **C_6** | 86 | 4.75 | 6.34 | 7.3 | N/A | I | no |
|  | **C_8** | 91 | 2 | 6.39 | 7.5 | N/A | IV | no |
|  | **C_9** | 81 | 2.75 | 6.59 | 7.9 | N/A | I | no |
|  | **C_10** | 79 | 2 | 6.91 | 6.4 | N/A | I | no |
|  | **C_11** | 63 | 1.5 | 6.60 | 7.8 | N/A | II | no |
|  | C_12 | 66 | 18.7 | 6.75 | 7.7 | N/A | N/A | no |
|  | C_13 | 69 | 15.3 | 7.32 | 8 | N/A | N/A | no |
|  | C_14 | 79 | 20.92 | 6.46 | 7.8 | N/A | N/A | no |
|  | C_16 | 58 | 20.16 | 6.69 | 7.5 | N/A | N/A | no |
|  | C_17 | 70 | 20.93 | 6.81 | 7.7 | N/A | I | no |
|  | C_18 | 66 | 16.97 | 6.75 | 7.8 | N/A | I | no |
|  | C_19 | 73 | 19.42 | 6.71 | 7.8 | N/A | I | no |
|  | C_20 | 60 | 24.23 | 6.87 | 8.5 | N/A | N/A | no |
|  | C_21 | 76 | 26.16 | 6.52 | 7.3 | N/A | I | no |
|  | C_22 | 61 | 17 | 6.79 | 8 | N/A | N/A | no |
|  | C_23 | 62 | 18.33 | 6.43 | 7.5 | N/A | I | no |
|  | C_24 | 69 | 25.92 | 6.63 | 7.1 | N/A | I | no |
|  | C_25 | 61 | 25 | 7.00 | 8.3 | N/A | I | no |
|  | C_26 | 88 | 11.06 | 6.36 | 6.7 | N/A | II | no |
|  | C_27 | 71 | 39.67 | 6.67 | 7.4 | N/A | I | no |
|  | C_29 | 93 | 12.9 | 6.55 | 4.8 | N/A | N/A | no |
| PD | **P_1** | 70 | 1.83 | 6.67 | 6.5 | 58 | III | yes |
|  | **P_3** | 74 | 3 | 6.47 | 6.4 | 72 | I | yes |
|  | P_4 | 70 | 2.33 | 6.58 | 8.3 | 30 | II | yes |
|  | P_5 | 77 | 1.66 | 6.43 | 6.2 | 64 | III | yes |
|  | **P_6** | 83 | 2 | 6.58 | 7.6 | 76 | III | no |
|  | **P_7** | 73 | 7.16 | 6.69 | 8 | 55 | II | no |
|  | **P_8** | 72 | 3.5 | 6.72 | 5.6 | 55 | II | no |
|  | P_9 | 69 | 4.16 | 6.66 | 8.3 | 68 | III | yes |
|  | P_11 | 77 | 1.16 | 6.59 | 6.4 | 55 | I | N/A |
|  | **P_12** | 80 | 2 | 6.54 | 8.2 | 69 | N/A | N/A |
|  | P_13 | 83 | 2.16 | 6.83 | 8.3 | 79 | III | N/A |
|  | P_14 | 80 | 2.25 | 6.65 | 8 | 55 | II | N/A |
|  | P_15 | 84 | 2.5 | 6.47 | 7.1 | 80 | II | N/A |
|  | P_16 | 88 | 2 | 6.71 | 8.4 | 85 | II | N/A |
|  | **P_18** | 81 | 2.5 | 6.62 | 6.4 | 73 | I | yes |
|  | **P_19** | 77 | 4 | 6.76 | 7 | 73 | III | yes |
|  | P_20 | 64 | 3.75 | 7.04 | 8.3 | 59 | II | yes |
|  | P_21 | 85 | 2.66 | 6.75 | 8.4 | 85 | III | yes |
|  | P_22 | 94 | 9.25 | 6.79 | 6.4 | N/A | III | no |
|  | P_24 | 67 | 8.25 | 6.67 | 7.9 | 58 | I | no |
|  | P_26 | 85 | 15.75 | 6.53 | 6.3 | 65 | II-III | no |
|  | P_27 | 75 | 6.67 | 6.93 | 8.2 | N/A | I | no |
|  | P_28 | 74 | 15.15 | 6.67 | 7.8 | N/A | II | no |
|  | P_29 | 89 | 30.75 | 6.67 | 7.2 | 72 | II-III | yes |
|  | P_30 | 66 | 11.21 | 6.73 | 7.9 | 55 | I | yes |
|  | P_31 | 65 | 7.83 | 7.13 | 6.9 | N/A | I | no |
|  | P_33 | 85 | 19.3 | 6.47 | 6.6 | 71 | N/A | N/A |

^1^Bold samples were used in the validation study.

^2^The information was derived from the available neuropathological reports; N/A = information was not available.
