# Supplementary material for: Gene Expression Profiles in Parkinson Disease Prefrontal Cortex Implicate FOXO1 and Genes under Its Transcriptional Regulation
Source: PLoS Genet. 2012 Jun 28;8(6):e1002794. doi: 10.1371/journal.pgen.1002794 (PMC3386245; doi:10.1371/journal.pgen.1002794)
Supplement: Table S3 — Validation study results. (DOC) [file pgen.1002794.s004.doc]

Supplementary Table 3. Validation study results.

| **Gene**  **(Microarray Probe)** | **Microarray p (fold change^1^)** | **Validation subset microarray p**  **(fold change^1^)** | **Validation p**  **(fold change^1^)** |
| --- | --- | --- | --- |
| *DDAH2*  (A_23_P19482) | 8.6E-5 (1.30) | 0.334 (1.12) | 0.222 (1.15) |
| *ELMO1**  (A_23_P122937) | 3.2E-4 (0.74) | 0.042 (0.71) | 0.120 (1.14) |
| *FOXO1*  (A_23_P151426,  A_24_P22079) | 3.7E-5 (1.51)  6.6E-5 (1.25) | 0.767 (1.06)  0.591 (1.04) | 0.035 (1.39) |
| *MAPRE1*  (A_24_P220058) | 4.6E-5 (1.33) | 0.280 (1.16) | 0.008 (1.29) |
| *NFE2L2*  (A_23_P5761) | 4.0E-4 (1.37) | 0.178 (1.29) | 0.007 (1.65) |
| *PDIA6*  (A_24_P319715) | 2.8E-4 (1.37) | 0.020 (1.72) | 0.011 (1.53) |
| *PEX12*  (A_24_P416411) | 6.8E-5 (1.26) | 0.003 (1.38) | 0.064 (1.33) |
| *RNF138*  (A_23_P89755) | 1.4E-4 (1.30) | 0.021 (1.39) | 0.006 (1.39) |
| *SEC24B*  (A_23_P69683) | 3.9E-4 (1.27) | 0.081 (1.33) | 0.096 (1.13) |
| *USPL1*  (A_24_P338757) | 5.0E-4 (1.28) | 0.005 (1.62) | 0.012 (1.46) |

^1^Fold change > 1 for increased expression in PD samples

*opposite direction of effect between the microarray and the validation studies
