# Supplementary material for: Gene Expression Profiles in Parkinson Disease Prefrontal Cortex Implicate FOXO1 and Genes under Its Transcriptional Regulation
Source: PLoS Genet. 2012 Jun 28;8(6):e1002794. doi: 10.1371/journal.pgen.1002794 (PMC3386245; doi:10.1371/journal.pgen.1002794)
Supplement: Table S4 — FOXO1 probe expression results for all Affymetrix substantia nigra PD microarrays available in the Array Express and National Brain Databank repositories1. (DOC) [file pgen.1002794.s005.doc]

**Supplementary Table 4. *FOXO1* probe expression results for all Affymetrix *substantia nigra* PD microarrays available in the Array Express and National Brain Databank repositories^1^**.

| **Array Type** | **Array Express ID** | **Used covariates** | **#PD/#C^2^** | ***FOXO1* probe 202723_s_at** | | ***FOXO1* probe 202724_s_at** | | ***FOXO1* probe**  **228484_s_at** | | **FoxO1 genes enrichment^4^ (FE/p)** |
| --- | --- | --- | --- | --- | --- | --- | --- | --- | --- | --- |
|  |  |  |  | **p** | **FC** | **p** | **FC** | **p** | **FC** |  |
| **Affymetrix**  **HG-U133A** | E-GEOD-8397 | no covariates  age, sex | 9/6 (LSN)  15/7 (MSN)  9/6 (LSN)  15/7 (MSN) | **0.047**  0.053  0.475  0.269 | 1.29  1.30  1.10  1.18 | **0.039**  **0.002**  **0.029**  **0.006** | 1.48  1.57  1.72  1.62 | N/A | N/A | 1.4/9.8E-14 (LSN)  1.4/2.2E-15 (MSN) |
|  | E-GEOD-20163 | no covariates  age | 8/9  4/8 | 0.396  0.076 | 0.86  1.17 | 0.585  0.790 | 1.06  1.04 | N/A | N/A | 1.3/6.7E-8 |
|  | E-GEOD-20164 | no covariates  age, sex | 6/5  6/5 | 0.389  0.699 | 1.38  1.19 | **0.021**  0.073 | 1.45  1.43 | N/A | N/A | 1.2/8.7E-3 |
|  | E-GEOD-20186 | no covariates  age | 14/14  10/13 | 0.850  0.302 | 1.04  1.33 | 0.580  0.439 | 1.35  1.67 | N/A | N/A | 1.4/2.9E-5 |
|  | E-GEOD-20295 | no covariates  age, sex | 11/15  11/15 | **0.002**  **0.003** | 1.53  1.56 | 0.537  0.722 | 1.14  1.08 | N/A | N/A | 1.4/5.1E-7 |
|  | National Brain Databank | no covariates  age, sex | 10/9  10/9 | 0.184  0.242 | 1.01  1.01 | 0.253  0.384 | 1.09  1.07 | N/A | N/A | p>0.05 |
| **Affymetrix**  **HG-U133_Plus_2** | E-GEOD-7621 | no covariates  sex | 16/9  16/9 | 0.228  **0.049** | 1.19  1.34 | 0.352  **0.025** | 1.15  1.37 | 0.400  0.557 | 1.00  1.00 | 1.3/7.1E-22 |
|  | E-GEOD-20141 | no covariates  N/A | 10/8  N/A | 0.225  N/A | 0.89  N/A | 0.818  N/A | 0.93  N/A | 0.037  N/A | 1.12  N/A | 1.3/2.1E-6 |
| **Affymetrix**  **HG-Focus** | E-GEOD-20333 | no covariates  age, sex | 6/6  6/6 | N/A | N/A | 0.275  0.081 | 0.86  0.74 | N/A | N/A | p>0.05 |
| **Affymetrix**  **HG-U133A** | E-GEOD-20295  (BA9, reference) | no covariates  age, sex | 14/16  14/15 | **1.9E-4**  **0.001** | 1.70  1.48 | **0.029**  0.159 | 2.41  1.68 | N/A | N/A | 1.4/2.9E-7 |
| **Weighted Z-score meta-analysis SN^3^** | **HG-U133A**  **HG-U133_Plus_2**  **HG-Focus** | no covariates  most available covariates | 96/82  88/80 | **7.4E-3**  **3.2E-4**  (N=8) | +  + | **4.1E-3**  **7.3E-3**  (N=9) | +  + | 0.023  0.036  (N=2) | +  + |  |

^1^The study E-GEOD-24378 was the only SN study available in Array Express, which was not included in the above table. This study (Affymetrix HG-U133_X3P array, did not have overlapping *FOXO1* probes with the Zhang et al. BA9 array; additionally, there is prior evidence that it behaves as an outlier when compared with other SN PD expression studies .

^2^LSN = lateral *substantia nigra*; MSN = medial *substantia nigra*

^3^The positive sign for the weighted Z-score meta-analysis results corresponds to increased expression in PD compared to control samples. No pooled effect size estimate is available when the weighted Z-score approach is used for meta-analysis. For E-GEOD-8397, the MSN data were used. N = number of studies included in the meta-analysis.

^4^The enrichment in FoxO1 TFBS genes was obtained in DAVID for the genes in each microarray experiment corresponding to probes with nominal p-values < 0.01 (the results were adjusted for sex and age covariates, if available). If the nominal p-value for enrichment was > 0.05, the FE and p values are not explicitly given (p>0.05). FE = fold enrichment
